# Effects of the lower energy and pulse stacking in carbon dioxide laser skin treatment: an objective analysis using second harmonic generation

**DOI:** 10.1590/ACB360304

**Published:** 2021-05-07

**Authors:** Marcos Matias Motta, Rafael Fantelli Stelini, Davi Reis Calderoni, Rovilson Gilioli, Gislaine Vieira Damiani, Carlos Lenz César, Paulo Kharmandayan

**Affiliations:** 1 Fellow PhD Degree. Universidade Estadual de Campinas – Faculty of Medical Sciences – Department of Surgery – Campinas (SP), Brazil.; 2 MD. Universidade Estadual de Campinas – Faculty of Medical Sciences – Department of Anatomic Pathology – Campinas (SP), Brazil.; 3 PhD. Universidade Estadual de Campinas – Faculty of Medical Sciences – Department of Surgery – Campinas (SP), Brazil.; 4 PhD, Head. Universidade Estadual de Campinas – Faculty of Medical Sciences – Multidisciplinary Center for Biological Investigation on Laboratory Animals Science – Campinas (SP), Brazil.; 5 PhD. Instituto Federal de Educação, Ciência e Tecnologia de São Paulo – Biophotonics Laboratory – Capivari (SP), Brazil.; 6 PhD. Universidade Estadual de Campinas – National Institute of Photonics Applied to Cell Biology – Biophotonics Laboratory – Campinas (SP), Brazil.; 7 Associate Professor, Head. Universidade Estadual de Campinas – Faculty of Medical Sciences – Department of Surgery – Campinas (SP), Brazil.

**Keywords:** Lasers, Gas, Skin Aging, Second Harmonic Generation Microscopy, Rats

## Abstract

**Purpose:**

To evaluate the effect of fractional carbon dioxide (CO_2_) laser treatment using lower power associated with pulse stacking within collagen fibers, using second harmonic generation microscopy and computerized image analysis.

**Methods:**

Twenty male Wistar rats aging eight weeks were used. Each treatment area received a single-pass CO_2_ fractional laser with different parameters. The 20 animals were divided into two groups and euthanized after 30 and 60 days. Second harmonic generation images were obtained and program ImageJ was utilized to evaluate the collagen organization within all areas. Collagen anisotropy, entropy and optical density were quantified.

**Results:**

Increased anisotropy over time was observed in all four areas, but only reached statistical significance (p = 0.0305) when the mildest parameters were used (area four). Entropy decreased over time in all areas, but without significance(p = 0.1779) in area four. Density showed an overtime increase only in area four, but no statistical significance was reached (p = 0.6534).

**Conclusions:**

When combined, the results obtained in this study regarding anisotropy, entropy and density tend to demonstrate that it is possible to achieve collagen remodeling with the use of lower power levels associated with stacked pulses.

## Introduction

Skin resurfacing with carbon dioxide (CO_2_) laser is still considered the gold standard treatment for facial rejuvenation. It has been used for this purpose since the early 90s with impressive results^1^,^2^. On the other hand, it presents a relatively high rate of drawbacks, as long downtime for recovery and risks for scarring and pigmentary disorders^3^.

Fractioning the laser beam with scanners was initially described by Manstein^4^ with a 1500 μm laser prototype. In 2007, Hantash^5^,^6^ described the CO_2_ fractional laser. Its principle relied on creating an array of multiple micro areas of tissue vaporization (micro thermal zones – MTZ) while leaving unaffected skin around them. This allowed for faster re-epithelization and recovery time while yielding good clinical results^7^,^8^.

Although much safer, the fractionated mode can still present some complications, especially when higher fluences are used, as observed by Shamsaldeen^9^.

An alternative for safer fractional CO_2_ laser treatments could be lowering the energy employed while stacking pulses at the same MTZ. This is already employed in clinical practice and usually delivers reliable results. An experimental study showed that using lower power associated with pulse stacking (consecutive pulses at the same location) can sustain higher macroscopic tissue contraction after 60 days compared to the use of high energy with a single pulse^10^.

In this study, we objectively evaluate this effect within collagen fibers using second harmonic generation (SHG) microscopy and computerized image analysis.

Collagen stands for the most abundant element of the extracellular matrix (ECM) and is responsible for maintaining skin tensile strength^11^,^12^. Due to its triple helix structure, which is not centrosymmetric, collagen is a very good SHG generator^12^–^14^ and the resultant images can be evaluated by computational analysis.

This study evaluated anisotropy, entropy and optical density. Anisotropy usually quantifies the degree of collagen fibers alignment within the dermis^15^ and can be used to study how it modifies, as skin ages or develops scars^16^. Entropy assesses the amount of disorderliness of a system and can be used to verify the randomness of an image. This was described previously for skin surface analysis^17^,^18^, as well as for the study of nerve aging^19^. Optical density is a well-known way to quantify the number of collagen fibers within an image^20^.

The aim of this experimental animal study is to use these collagen features obtained from SHG images in order to evaluate the dermal effects of using lower CO_2_ laser power associated with pulse stacking.

## Methods

The study was approved by the board of the Ethical Committee of Animal Research (protocol #3012-1).

**Figure 1:**
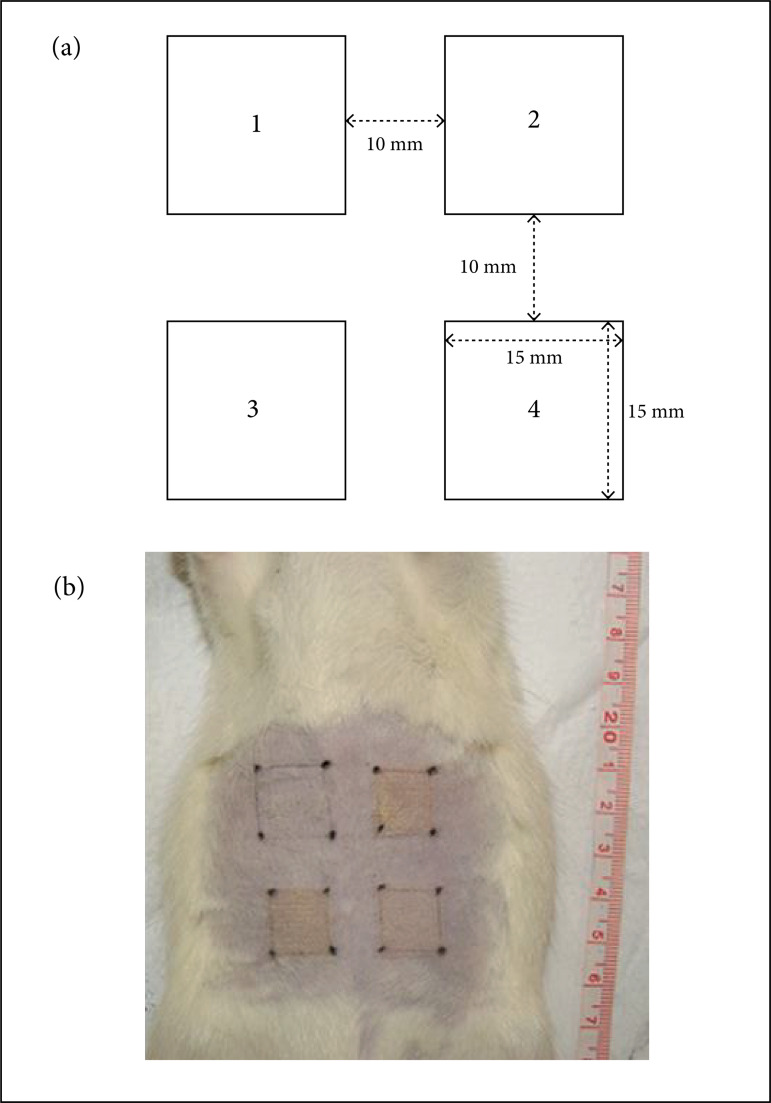
Treatment areas. **(a)** Dimensions and arrangement; **(b)** Areas immediately after laser application.

Twenty male Wistar rats aging eight weeks were used. They were kept on a 12-hour light/dark cycle with free access to water and standard laboratory chow (3,100 kcal·kg^–1^).All animals were anesthetized with 80 mg·kg^–1^ ketamine plus 10 mg·kg^–1^ xylazine injected intraperitoneally and positioned on dorsal decubitus. Their abdomens were shaved and stamped with four 15 × 15 mm squares, 10 mmapart from each other to assure that one treatment area does not influence others. Then, the vertices of each square were tattooed for later area identification.Each square was assigned a number from one to four, as seen in the diagram (Fig. 1). Areas two, three and four were defined as treatment areas and area one was the control.

### Groups for analysis

The 20 animals were divided into two groups.

Group 1 (n = 10) was sacrificed by anesthesia overdose 30 days after laser irradiation.

Group 2 (n = 10) was sacrificed 60 days post-procedure.

Each animal had all previously demarcated four areas collected for histological analysis.

Tissue specimens from both groups were fixed in 10% buffered formalin and embedded in paraffin. Then, a vertically cut 5 μm slice was obtained from each area and stained using the hematoxylin and eosin method.

### Laser procedure

Each treatment area received a single-pass CO_2_ fractional laser (Smartxide Dot; DEKA, Florence, Italy) with different parameters.

The 120 μm spot size was used, as well as a 500 μm spacing between MTZs for all areas in this study.

Other parameters, such as power, exposure time, stacking, fluence and energy per each MTZ, are summarized in Table 1.

**Table 1:** Laser settings for each area.

Area	Power (W)	Exposure time (μs)	Stack	Fluence (J·cm^–2^)	Energy/MTZ (mJ)
1	-	-	-	-	-
2	30	900	1	3.74	27.0
3	20	300	3	2.49	18.0
4	10	300	3	1.87	13.5

### Second harmonic generation image acquisition

**Figure 2:**
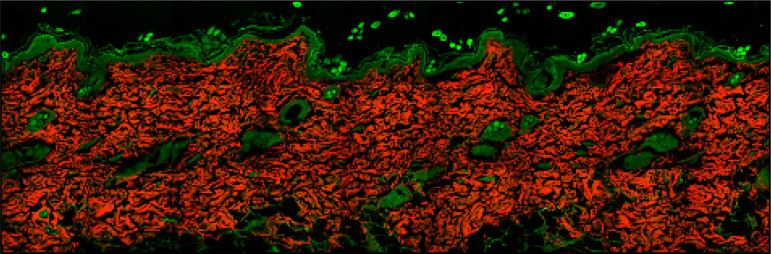
Second harmonic generation scans stitched in mosaic.

Second harmonic generation images were utilized to evaluate the collagen organization within all four areas. Images were acquired with an inverted Z.1 Axio Observer microscope equipped with a Zeiss LSM780 NLO confocal scanning head (Carl Zeiss AG, Jena, Germany) at the National Institute of Photonics Applied to Cell Biology. All samples were evaluated according to a protocol previously described by Utino^21^. To obtain a complete image of the slide, we acquired tile scans (512 × 512) that were stitched in larger mosaics, as seen in Fig. 2.

### Image evaluation

All images were analyzed using the free software ImageJ (National Institutes of Health, USA. http://www.imagej.nih.gov/ij)for collagen morphometric features. Collagen anisotropy, entropy and optical density were quantified. For each of these collagen features, a specific software plug-in is needed. All of them are already previously described for collagen analysis.

In order to measure anisotropy, we used the FibrilTool plug-in applied without any image pre-processing, as indicated by Boudaoud^22^.

Either for quantifying optical density and entropy, all images were split into color channels to obtain only the red channel, which is specific for the SHG signal.

To quantify collagen optical density, we clicked the “measurement” button under the “analyze” menu and the results were presented in the “results” window.

For entropy analysis, we used the grey level co-occurrence matrix (GLCM) texture analysis plug-in.

Each whole image was measured three times for each feature and averaged.

All data were tabulated on a sheet for further statistical analysis.

### Statistics

The SAS system was used for statistical analysis. Areas within a group were compared using the Friedman test, while intergroup comparison was conducted by the Mann–Whitney test. The variables studied were anisotropy, density and entropy. The level of significance used in this study was 5%.

## Results

All images were assessed for three collagen features: anisotropy, entropy and density.

### Anisotropy

In group 1 (30 days), all treatment areas showed decreased anisotropy when compared to the control area, but without statistical significance (p = 0.7891).

**Figure 3:**
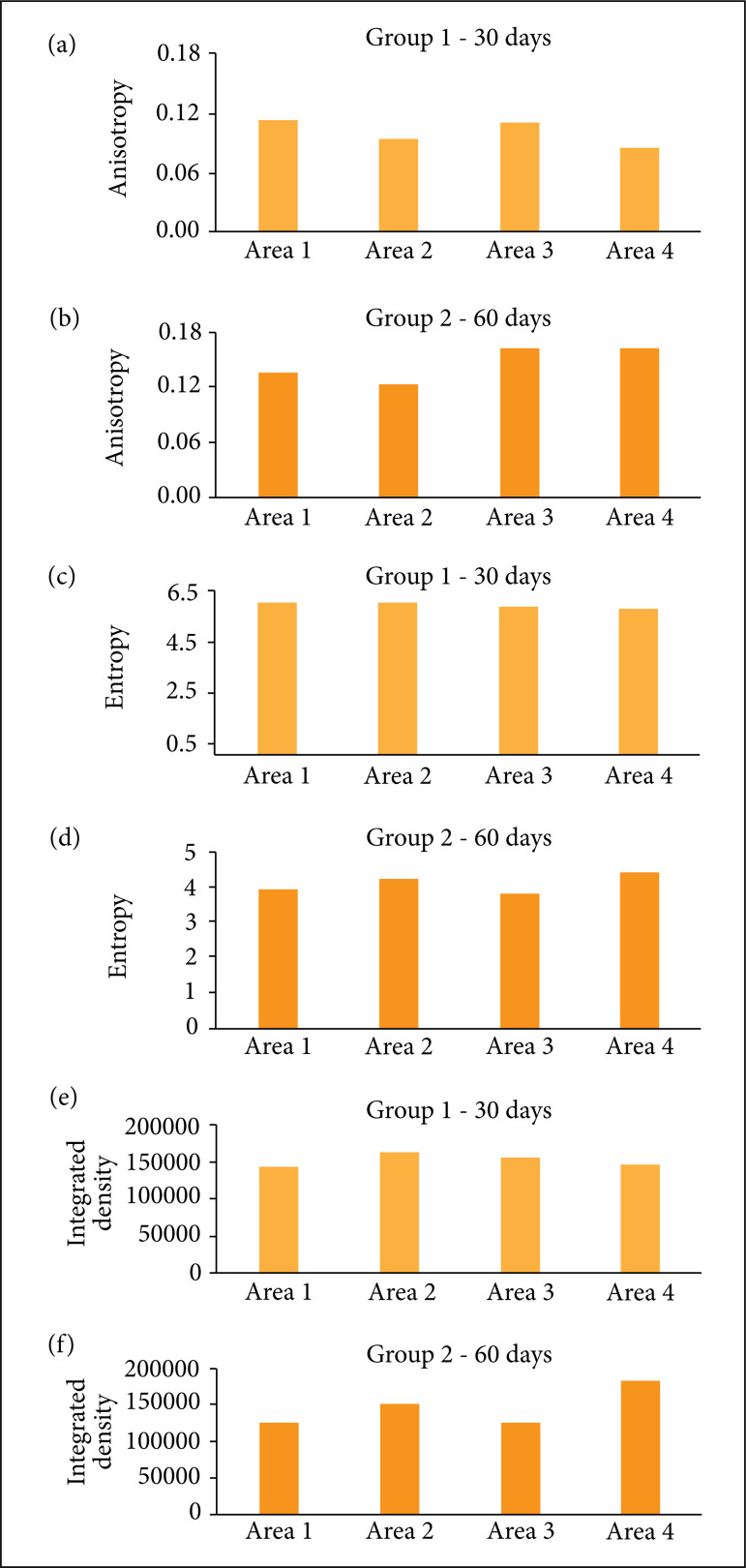
**(a)** Anisotropy results for group 1; **(b)** Anisotropy results for group 2; **(c)** Entropy results for group 1; **(d)** Entropy results for group 2; **(e)** Integrated density results for group 1; **(f)** Integrated density results for group 1.

In turn, in group 2 (60 days), we observed an increased anisotropy for treatment areas three and four when compared to the control area. On the other hand, anisotropy values were lower in the treatment area two than in the control area. Again, no statistical significance was found (p = 0.7014). These results are shown in Fig. 3.

When comparing areas between the two groups, we show an increase in anisotropy overtime for all of them, including the control area. This increase was more evident in areas three and four, but only reached statistical significance in area four (p = 0.0305).

**Figure 4:**
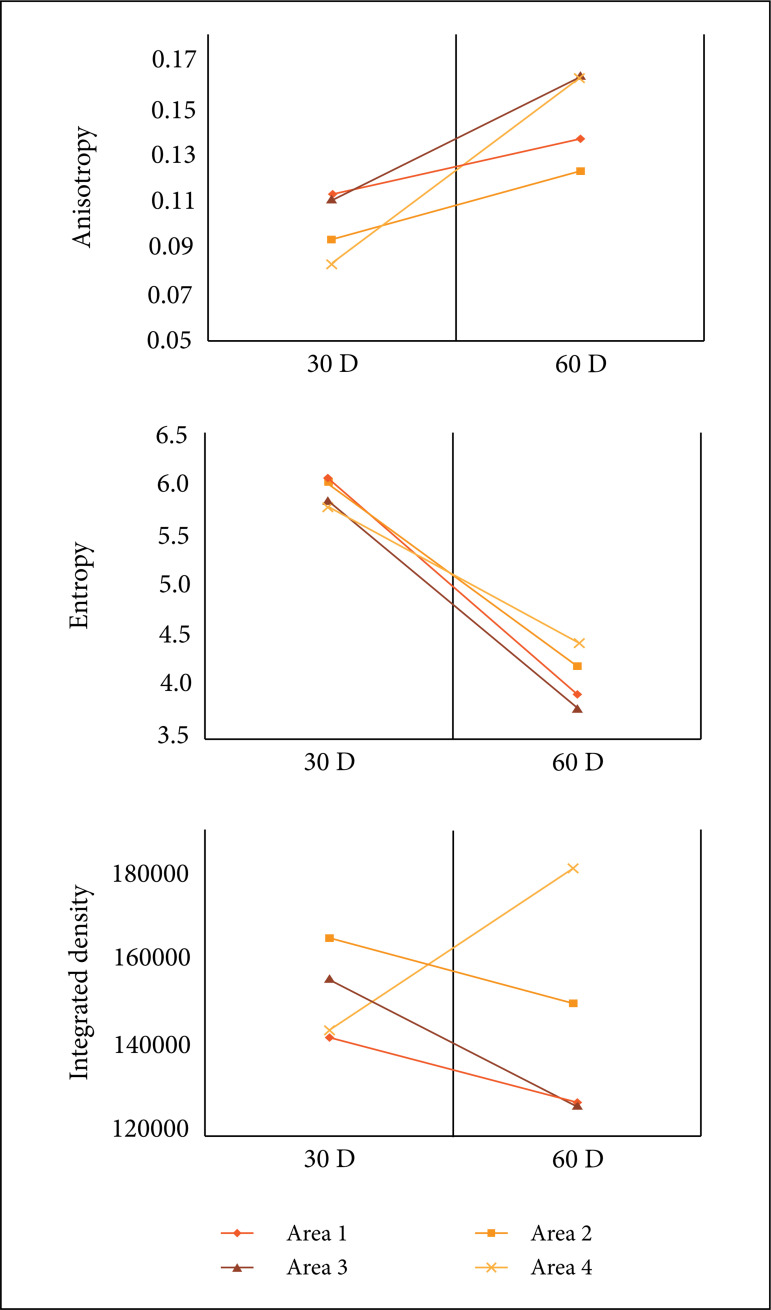
Collagen features behavior over time.

The results for anisotropy comparison between the two groups studied are represented in Fig. 4.

### Entropy

In group 1, there were no statistical differences between treatment areas and the control area (p = 0.8497). The same pattern was observed in group 2 (p = 0.4551). These results are demonstrated in Fig. 3.

However, when comparing the groups, we noticed an overtime decrease in entropy for all areas. This decrease showed statistical significance for the control area(p = 0.004), area two (p = 0.0113) and area three (p = 0.0013). Area four also showed a decrease in entropy, but without statistical significance (p = 0.1779). These results are demonstrated in Fig. 4.

### Density

In group 1, treatment areas two and three showed a slight increase in density compared to the control area. On the other hand, the treatment area four values were lower than the control area. However, no statistical significance was reached when comparing all areas (p = 0.4551) (Fig. 3).

Group 2 demonstrated that all treatment areas increased density against the control, especially treatment area four. Despite this major increase in density in the treatment area four, no statistical significance was reached when comparing areas (p = 0.2059) (Fig. 3).

In intergroup comparison, we observed a decrease in density in the control area (p = 0.9674) and treatment areas two (p = 0.6232) and three (p = 0.7337). Treatment area four, in its turn, showed an overtime increased density (p = 0.6534). No statistical significance was reached (Fig. 4).

## Discussion

Our study investigated the possibility of using lower power whereas associating pulse stacking to achieve collagen remodeling. For this purpose, we analyzed SHG images with ImageJ free software, quantifying three collagen features: anisotropy, entropy and density. Together, they enable a structural evaluation of collagen changes after fractional CO_2_ laser treatment.

Since the early 90s, the CO_2_ laser has evolved to become the gold standard for facial resurfacing^1^,^2^. It is possible to achieve impressive results in a single session due to its capability of skin contraction e collagen remodeling^23^. However, the long downtime for recovery, the risks for pigmentary disorders and unaesthetic scars rendered CO_2_ laser resurfacing a less useful tool for facial rejuvenation^3^.

The concept of fractional lasers emerged in 2004^4^ and its applicability to the CO_2_ laser was described by Hantash^5^,^6^. Since then, many authors have studied its ability to successfully treat photoaging^7^,^8^,^24^,^25^, as well as other disorders like hypertrophic scars^26^,^27^ and acne scars^28^. The idea of deep ablating dermal tissue whereas leaving intact surrounding skin made it possible to deliver an excellent result while reducing significantly the risks and downtime^5^,^29^. However, achieving deep ablation usually requires higher power levels^30^. Despite its superior side effect profile over full ablative CO_2_ lasers, the fractional mode still presents some drawbacks, especially when higher power levels are used^9^. Avram^31^ advised for the risks of hypertrophic scaring when fractional CO_2_ laser resurfacing is used on the neck.

In the search for reducing, even more, the overall risk profile of fractional CO_2_ laser, an option could be reducing power levels while increasing the number of pulses delivered to each MTZ (stacking). Pulse stacking has been studied since long before the advent of the fractional laser. Fitzpatrick^32^, using a full ablative CO_2_ laser, found a greater potential for scaring due to the increased thermal injury observed with pulse stacking. On the other hand, when using a fractional CO_2_ laser, the use of consecutive pulses seems to be beneficial. Oni^33^ demonstrated that, by doubling a pulse using half the energy, the tissue does not dissipate heat between pulses. It results in deeper and narrower columns of ablation with relatively wider zones of coagulation. This pattern could yield good results with less downtime than single pulses.

To investigate this concept, a previous study from our group^10^ concluded that it is possible to achieve similar MTZ dimensions with lower power and pulse stacking. The same study showed increased tissue contraction overtime when these less aggressive parameters had been used. Prignano^34^, studying cytokine responses in tissue remodeling, also demonstrates that it is possible to achieve good biological results using lower power levels.

In order to corroborate our previous results, in this present experimental study we aimed to histologically characterize changes in collagen structure that could confirm the effects of using lower power and pulse stacking in collagen remodeling.

There are many methods to evaluate collagen within the dermis, including electron microscopy, biochemical and immunohistochemical analysis, among others^35^. A study conducted by Reilly^36^ demonstrated that the molecular effects of both fractional and fully ablative CO_2_ laser are very similar. This could explain the consistent rejuvenation obtained with the fractional mode. Other authors examined collagen behavior after CO_2_ exposure employing specific stains^37^ or even by describing collagen changes in simple hematoxylin-eosin stains^38^. These methods, although largely used, many times are subject to subjective analysis^14^.

To overcome subjectivity, during the last decade several optical methods also had been developed, as confocal laser scanning, optical coherence tomography, and multiphoton microscopy, especially SHG microscopy^39^. All these methods provide images that can be further analyzed by image software, resulting in a more objective way to assess collagen behavior^40^.

In this study, SHG microscopy was employed to analyze collagen response to a fractional CO_2_ laser single treatment. Due to the collagen non-centrosymmetric molecular structure, it is an effective SHG generator^41^. The images obtained from SHG can be used to provide information on collagen structure within the skin^42^. Guo^43^ used SHG to investigate skin rejuvenation after treatment with a 1550 μm fractional laser. They concluded that it is an appropriate technique to evaluate collagen regeneration by the fractional laser treatment. The same study observed that lower power treatment associated with a higher density of MTZ induces faster collagen regeneration.

The anisotropy index quantifies the degree of preferred alignment of collagen fibrils within the dermis^44^. This is important for understanding skin behavior. In this study, anisotropy was quantified using a plug-in from ImageJ software named FibrilTool. It was designed based on the concept of nematic tensor from the physics of liquid crystals to measure how well collagen fibrils are aligned. This enables image analysis without the complex processing needed with other techniques. Values obtained vary from 0 (random orientation – isotropic) to 1 (total alignment – anisotropic)^22^.

As skin ages, collagen fibers become more parallel^16^,^45^, and the same happens to the scar tissue^14^. This results in higher anisotropy values. Interestingly, the same pattern is present in newly formed collagen after photothermal treatments as observed by Wu^44^. Thus, we expected an increase in anisotropy for the laser-treated areas as indicative of neocollagenesis. We did not observe this pattern 30 days after treatment. In fact, a decreased anisotropy compared to the control area was noted. Dainichi^46^ also observed that an increased collagen alignment was present only after 35 to 58 days from laser treatment due to the initial collagen degeneration. This parallel arrangement of collagen fibers was also observed *in vivo* 90 days after fractional CO_2_ laser treatment using confocal microscopy^47^.

However, after 60 days, the two areas treated with lower power and pulse stacking increased anisotropy compared to the control area. This pattern was not observed in the area treated with the highest power in a single pulse. Although these results did not reach statistical significance, they could indicate that milder parameters can stimulate collagen synthesis, as observed by Prignano^34^.

We also compared anisotropy of all areas between 30 and 60 days. All areas including the control area increased anisotropy overtime. Concerning the control area, the increase, although not significant, is expected due to collagen alignment seen with aging^16^. The laser-treated areas experienced a greater increase in anisotropy from 30 to 60 days, but it only reached statistical significance(p = 0.0305) in the area treated with the mildest parameters. We hypothesize that these findings were detected only 60 days after laser treatment due to the prolonged effect on collagen structure seen after fractional laser treatments. These long-term effects have been demonstrated immunohistochemically to last for up to six months^6^,^48^. Our results show a greater increase in anisotropy using less power and pulse stacking are in line with other authors. Yuan *et al*.^28^ demonstrated that lower fluences can provide a significant efficacy for acne scars whereas Prignano^38^ found no advantage, both clinically and histologically, to use treatment with 4.15 J·cm^–2^ laser irradiation compared to those obtained with 2.07 and 2.77 J·cm^–2^.

Entropy is one of the features of the textural analysis, known as the GLCM, an effective method for quantitative analysis of skin texture^49^. It can be used to assess the level of randomness of a digital image^18^ and has been associated with the degree of fiber organization^21^. We used entropy to quantify collagen structure behavior in the SHG images obtained after CO_2_ fractional laser irradiation. With aging, the skin (and consequently collagen fiber arrangement) loses randomness, and entropy values decrease^19^. In this way, CO_2_ fractional laser treatment ideally should slow down this collagen arrangement loss of complexity. Analyzing entropy 30 days after laser irradiation, we noticed almost no differences among all areas. In turn, after 60 days, it was possible to observe an increased entropy, although not statistically significant, for the treatment areas two and four. These are, respectively,the areas where the highest and the lowest laser parameters were employed. Important data was found when we analyzed entropy from a temporal perspective. From 30 to 60 days after laser treatment, all areas including the control area showed decreased entropies. These results confirm the observations of Silva^19^, who demonstrated a loss of randomness with aging. The only area in which the entropy decrease was not statistically significant(p = 0.1779) was the area where de mildest parameters were employed. This result could indicate that by using lower power and associating stacked pulses, it is possible to slow down entropy decline overtime. To our knowledge, no previous study has evaluated collagen entropy on SHG images after fractional CO_2_ laser irradiation.

Integrated density is one of the most used parameters in SHG image analysis and an excellent form of collagen quantification. Its measures (the product of area and mean gray level) can be associated with the number of collagen fibers^20^. Collagen density decreases with aging, as it has been demonstrated both using regular stains^50^ and SHG microscopy^51^. In our study, almost all treated areas showed an increased collagen density when compared to the control area, but without statistical significance. But when density was analyzed over time, we observed that only the area where the mildest parameters were employed (area four) had an increase, whereas all the other areas showed the expected decreased density with aging^39^,^51^. These results also did not reach statistical significance, probably due to the small sample studied. Nevertheless, it still might indicate that it is possible to induce neocollagenesis using less aggressive laser settings.

It has been already demonstrated that fractional CO_2_ laser is clinically effective^52^. Tierney^53^ showed a 65.3% mean clinical improvement in lower lid laxity six months after two to three treatments. Another study with the same equipment used in this paper found a 52.4% mean improvement for overall cosmetic outcome after two to three sessions treating moderate to severe photoaging^54^. However, most of these studies show some degree of subjectivity when evaluating the results obtained.

Attempts to relate the clinical effects obtained with the application of fractional CO_2_ laser to changes in the dermis were also carried out with different methods. For example, Ozog^37^ used the Herovici stain to differentiate types I and III collagen using computation analysis of digital images evaluated after treating mature burn scars. Prignano^38^ provided a clinical to histological correlation using hematoxylin-eosin stains after a single treatment with a fractional CO_2_ laser device. Even the molecular mechanisms underlying fractional CO_2_ laser clinical outcomes were already studied^36^. The use of SHG signal to evaluate collagen responses to laser treatments was already studied by some authors^43^,^44^.

Our study, in its turn, uses SHG images to objectively evaluate collagen changes after fractional CO_2_ laser irradiation with lower power and pulse stacking. Although not conclusive, our results show a tendency that is in line with other authors^33^,^38^. Besides that, our findings corroborated our previous study^10^ on the macroscopic skin tightening observed using the same concept.

Further studies should investigate this theory within the human dermis to determine ideal laser settings balancing good results and low risks. In this way, SHG microscopy provides quantifiable measures for evaluation and can also be applied *in vivo* without the need for skin biopsies.

## Conclusion

The combined results regarding anisotropy, entropy and density tend to demonstrate that it is possible to achieve collagen remodeling with the use of lower power levels associated with stacked pulses.
